# Pain and Biopsychosocial Features in Patients With Myogenous Temporomandibular Disorders: A Retrospective Study According to the DC/TMD Axis II

**DOI:** 10.1111/odi.70126

**Published:** 2025-10-29

**Authors:** Martina Ferrillo, Nicola Marotta, Andrea Demeco, Laura Gallelli, Maria Teresa Inzitari, Umile Giuseppe Longo, Francesco Riccitiello, Antonio Ammendolia, Amerigo Giudice, Alessandro de Sire

**Affiliations:** ^1^ Dentistry Unit, Department of Health Sciences University of Catanzaro “Magna Graecia” Catanzaro Italy; ^2^ Physical Medicine and Rehabilitation Unit, Department of Experimental and Clinical Medicine University of Catanzaro “Magna Graecia” Catanzaro Italy; ^3^ Research Center on Musculoskeletal Health, MusculoSkeletalHealth@UMG University of Catanzaro “Magna Graecia” Catanzaro Italy; ^4^ Physical Medicine and Rehabilitation Unit, Department of Medical and Surgical Sciences University of Catanzaro “Magna Graecia” Catanzaro Italy; ^5^ Fondazione Policlinico Universitario Campus Bio‐Medico Roma Italy; ^6^ Research Unit of Orthopaedic and Trauma Surgery, Department of Medicine and Surgery Università Campus Bio‐Medico di Roma Roma Italy; ^7^ Department of Neuroscience, Reproductive Sciences and Dentistry University of Naples Federico II Naples Italy

**Keywords:** biopsychosocial model, musculoskeletal pain, orofacial pain, pain‐related impairment, psychosocial assessment, temporomandibular disorders

## Abstract

**Objectives:**

This study aimed to search for a correlation between myogenous TMD diagnosis and Axis II assessment, and to identify the psychosocial predictors of high pain‐related disability.

**Material and Methods:**

In this retrospective study, myogenous TMD patients referring to a University Hospital were included. TMD symptoms were assessed by DC/TMD and validated screening tools for TMD pain. Psychosocial status and pain‐related disability were assessed by means of the Patient Health Questionnaire‐15 (PHQ‐15), Jaw Functional Limitation Scale‐20 (JFLS‐20), Generalized Anxiety Disorder‐7 (GAD‐7), and Graded Chronic Pain Scale Version 2.0 (GCPS).

**Results:**

The final cohort involved 73 TMD patients and 73 controls. The GCPS CPI and JFLS‐20 global score were significantly different in TMD patients (all subtypes) compared to controls (all *p* < 0.001). GCPS CPI was significantly different in local myalgia compared to either myofascial pain or with referral (all *p* < 0.001). In terms of PHQ‐15, no significant differences were reported in local myalgia compared to controls (*p* = 0.996). GAD‐7 did not differ among groups (all *p* > 0.05).

**Conclusions:**

Findings from the present study revealed that myofascial pain patients reported significantly higher levels of pain intensity, functional limitations, and physical symptoms, compared to both local myalgia and control patients.

## 1 Introduction

“Temporomandibular disorders (TMD)” is an umbrella term covering a set of musculoskeletal conditions related to the masticatory musculature, temporomandibular joints (TMJ), and surrounding structures (Alomar et al. 2007; Schiffman et al. 2014). TMD affect approximately 31% of the adult and 11% of the pediatric population and are considered the main cause of non‐dental pain in the orofacial region, and the second most common musculoskeletal disorders causing chronic pain and disability (Valesan et al. 2021).

The Diagnostic Criteria for Temporomandibular Disorders (DC/TMD) were established to allow clinicians to perform a complete clinical evaluation through a dual‐axis system composed of Axis I for physical assessment and Axis II for psychosocial evaluation (Schiffman et al. 2014). The Axis II evaluates the psychosocial status and the pain‐related disability, considering the biopsychosocial features that could be affected by TMD, such as emotional and cognitive balance, sleep, social and physical activities, leading to higher levels of anxiety and lower quality of life (Resende et al. 2020). The etiology of TMD is considered complex and multifactorial and is influenced by a combination of biological and psychosocial factors, encompassing genetics, oral behaviors, poor general health and somatic symptoms, sex hormones, and psychological distress (Shaefer et al. 2013; Ohrbach et al. 2011).

The main strength of the DC/TMD Axis II lies in the importance given to the evaluation of pain‐related disability. In fact, psychosocial issues, such as perceived stress, previous life events, negative affect, depression, somatic symptoms, and mood are known to be important predictors for TMD pain onset (Ohrbach et al. 2011; Yap et al. 2002). In addition, biopsychosocial risk factors, such as a high level of depression, anxiety, and non‐specific physical symptoms were linked with pain levels in these patients (Canales et al. 2019; Manfredini, Winocur, et al. 2010).

The association among psychological distress, high prevalence of pain‐related disability, and increased TMD pain was corroborated in previous studies (Manfredini, Winocur, et al. 2010; Manfredini, Borella, et al. 2010; Manfredini et al. 2011), with somatic awareness and depression as common features among patients who suffer from persistent TMD pain (Manfredini, Borella, et al. 2010).

Moreover, self‐perceived poor general health, increased pain‐related worry, subjective illness perceptions, as well as comorbid pains are associated with the complexity of TMD pain and considered risk factors for the development of chronic pain (Manfredini et al. 2011; Galli et al. 2010) and predictors of treatment outcome (Dworkin et al. 2002).

Considering the above, this study was performed on TMD data from a Southern Italy cohort with the aim to: (I) search for a correlation between myogenous TMD diagnosis and Axis II assessment; (II) identify the psychosocial predictors of high pain‐related disability, whose assessment should be useful during the clinical decision‐making processes.

## 2 Material and Methods

### 2.1 Participants

In this retrospective study, we included data of adult subjects to the Gnathology Unit of the University Hospital “Renato Dulbecco” of Catanzaro, Italy, in an 18‐month period from January 2023 to June 2024. We considered for inclusion patients with a diagnosis of myalgia, including local myalgia, myofascial pain, and myofascial pain with referral subtypes (Schiffman et al. 2014). The clinical examination was performed according to the DC/TMD Axis I and was based on the DC/TMD Demographic Questionnaire, DC/TMD Symptom Questionnaire, TMD Pain Screener, DC/TMD Examination Form, and Axis I decision trees (Schiffman et al. 2014).

Exclusion criteria were: (1) previous or concomitant treatment for temporomandibular disorders (e.g., occlusal splint); (2) concurrent illness, including neoplastic, coagulopathic, neurological, vestibular, visual, or psychiatric disorders; (3) history of traumatic cervical spine or temporomandibular injury or head–neck surgery; (4) patients with congenital abnormality or neoplastic conditions in the TMJ region; (5) children or adolescents during growth; (6) concomitant therapy with analgesic drugs or rehabilitation therapies (e.g., physical therapy) for orofacial or neck pain.

Controls were recruited among the patients' companions in the waiting room of the dental school of the University Hospital. Subjects who agreed to participate completed the questionnaires and underwent the clinical examination according to the DC/TMD Axis I, and only subjects without TMD diagnosis were considered for inclusion. Subjects were matched for age and sex, and the same inclusion and exclusion criteria were applied to the control group (except for the TMD diagnosis).

The study was approved by the Ethical Committee of Calabria Region (protocol number: 61/2025). All participants were asked to carefully read and sign an informed consent, and researchers provided to protect the privacy and the study procedures according to the Declaration of Helsinki, with pertinent National and International regulatory requirements. Moreover, the study was performed in accordance with the STrengthening the Reporting of OBservational studies in Epidemiology (STROBE) Guidelines.

### 2.2 DC/TMD Axis II Assessment

Psychosocial status and pain‐related disability were assessed according to the DC/TMD Axis II by evaluating relevant behavior, psychological status, and psychosocial functioning (Galli et al. 2010). The DC/TMD Axis II consisted of the following self‐administered questionnaires: the Graded Chronic Pain Scale Version 2.0 (GCPS) (Von Korff et al. 1992), the Jaw Functional Limitation Scale‐20 (JFLS‐20) (Ohrbach et al. 2008), the General Anxiety Disorder‐7 (GAD‐7) (Spitzer et al. 2006), and the Patient Health Questionnaire‐15 (PHQ‐15) (Kroenke et al. 2002).

### 2.3 TMD Pain

A Visual Analogue Scale (VAS) was used to evaluate the intensity of pain in terms of TMD pain perceived by the patient through a self‐administered score ranging from 0 (no pain) to 10 (pain as bad as could be). Specifically, we assessed:

– the current pain VAS asking to the patient: “How would you rate your facial pain right now?”;
– the worst pain VAS asking to the patient: “In the last 30 days, on average, how would you rate your worst facial pain?”;
– the average pain VAS asking to the patient: “In the last 30 days, on average, how would you rate your facial pain?”.

### 2.4 Neck Pain

The number of subjects who reported having neck pain was collected through a self‐administered yes/no question: “In the last 30 days, have you experienced neck pain?”

### 2.5 Statistical Analysis

Data management and analyses were performed via a pre‐specified statistical analytical design; thus, statistical analysis was conducted using the R‐4.4.1 software (R Foundation, Vienna, Austria). The continuous variables are presented as means ± standard deviations. Regarding the comparison of means of two or more samples as significantly different, we have performed one‐way analysis of variance (or one‐way ANOVA).

Afterwards, we performed a machine learning approach by sequentially adding predictors to a decision tree ensemble, each one correcting its predecessor (Yoon et al. 2018; Polikar 2006). Nevertheless, instead of shifting the weights for every incorrect ranked observation at every node, the boosting method suited each new feature to the residual errors assembled by the previous one (Pinto et al. 2022). For each model, data were split into a training set, which consisted of a random subset defining 80% of the data and a holdout set incorporating the remaining 20% (Joseph and Vakayil 2021).

We performed a random forest model exploiting fewer variables and omitting features during the calculations to change the defined variance, determining the intrinsic effect of including or excluding the variables (Marotta et al. 2022).

So, the outcome is a rank described as a variable influence. In summary, the boosting classification is a machine learning model that assesses not only the importance of variables but also how best or worst the prediction would be if one or more variables were removed, shielding the elimination of good predictor variables (Ferrillo et al. 2022).

Lastly, we performed a decision tree model to estimate within the considered variables at which scores we could discriminate a patient profile with TMD. In this scenario, the decision tree model is a classification method that constructs a set of decision trees consisting of a large number of trees, operating as an ensemble. Each tree returns a class prediction in the random forest, and the class with the most ranks becomes the model's prediction.

## 3 Results

Out of 104 consecutive TMD patients screened for eligibility, 73 patients (25 males and 48 females, mean age 41.38 ± 16.25 years) were included. The number of female patients was 1.7 times that of male patients and the median age of patients was 39.00 years. Patient demographic and clinical characteristics are listed in Table 1.

**TABLE 1:** Demographic characteristics of the subjects included in the study (*n* = 146).

	TMD patients (*n* = 73)	TMD patients with local myalgia (*n* = 29)	TMD patients with myofascial pain (*n* = 26)	TMD patients with myofascial pain with referral (*n* = 18)	Controls (*n* = 73)
Age (years)	41.38 ± 16.25	40.79 ± 17.27	40.15 ± 16.73	44.11 ± 14.30	41.38 ± 16.25
*Gender*					
Male (*n*, %)	25 (34.25)	15 (51.73)	4 (15.38)	6 (33.34)	25 (34.25)
Female (*n*, %)	48 (65.75)	14 (48.27)	22 (84.62)	12 (66.66)	48 (65.75)
*Educational level*					
Elementary school (*n*, %)	2 (2.74)	1 (3.45)	0 (0.00)	1 (5.55)	0 (0.00)
Junior high school (*n*, %)	14 (19.18)	6 (20.69)	6 (23.08)	2 (11.11)	4 (5.48)
High school (*n*, %)	34 (46.58)	12 (41.38)	13 (50.00)	9 (50.0)	30 (41.10)
Master degree (*n*, %)	23 (31.50)	10 (34.48)	7 (26.92)	6 (33.34)	39 (53.42)

*Note:* Continuous variables are expressed as means ± standard deviations; categorical variables are expressed as percentages.

According to DC/TMD diagnosis, 29 patients (39.71%) (15 males and 14 females, mean aged 40.79 ± 17.27) were diagnosed with local myalgia, 26 patients (35.62%) (4 males and 22 females, mean aged 40.15 ± 16.73) with myofascial pain, and 18 patients (24.67%) (6 males and 12 females, mean aged 44.11 ± 14.30) with myofascial pain with referral.

The control group consisted of 73 subjects (25 males and 48 females, mean age 41.38 ± 16.25 years) with no TMD diagnosis.

Demographic characteristics such as age, gender, and educational level are reported in Table 1.

TMD diagnosis according to the DC/TMD is reported in Table 2. Interesting to note, 50 patients (68.49%) reported disc displacement with reduction, and 25 (34.25%) reported headaches attributed to TMD. Moreover, 51.73% of TMD patients with local myalgia, 73.08% of patients with myofascial pain, and 72.22% of patients with myofascial pain with referral reported arthralgia.

**TABLE 2:** Temporomandibular disorders diagnosis according to the Diagnostic Criteria for temporomandibular disorders.

	TMD patients (*n* = 73)	TMD patients with local myalgia (*n* = 29)	TMD patients with myofascial pain (*n* = 26)	TMD patients with myofascial pain with referral (*n* = 18)
Arthralgia (*n*, %)	47 (64.38)	15 (51.73)	19 (73.08)	13 (72.22)
Headache attributed to TMD (*n*, %)	25 (34.25)	1 (3.45)	14 (53.85)	10 (55.55)
Disc displacement with reduction (*n*, %)	50 (68.49)	19 (65.52)	16 (61.54)	15 (83.33)
Disc displacement with reduction, with intermittent locking (*n*, %)	1 (1.37)	0 (0.00)	1 (3.85)	0 (0.00)
Disc displacement without reduction, with limited opening (*n*, %)	3 (4.11)	0 (0.00)	0 (0.00)	3 (16.67)
Disc displacement without reduction, without limited opening (*n*, %)	1 (1.37)	0 (0.00)	1 (3.85)	0 (0.00)
Degenerative Joint disease (*n*, %)	8 (10.96)	2 (6.89)	4 (15.38)	2 (11.11)
Subluxation (*n*, %)	5 (6.85)	2 (6.89)	2 (7.69)	1 (5.55)

*Note:* Categorical variables are expressed as percentages.

The prevalence of TMD symptoms and psychosocial status is reported in Table 3.

**TABLE 3:** Psychosocial status, pain‐related disability, and oral behaviors of the subjects included in the study (*n* = 146).

	TMD patients (*n* = 73)	TMD patients with local myalgia (*n* = 29)	TMD patients with myofascial pain (*n* = 26)	TMD patients with myofascial pain with referral (*n* = 18)	Controls (*n* = 73)
GCPS CPI	50.62 ± 26.22	32.03 ± 18.85	59.08 ± 22.00	68.33 ± 24.17	0.75 ± 3.32
GCPS pain days	83.30 ± 72.38	52.59 ± 63.51	96.04 ± 65.50	114.39 ± 79.71	0.18 ± 0.82
GCPS disability days points	1.16 ± 1.42	0.31 ± 0.85	1.65 ± 1.50	1.83 ± 1.42	0.00 ± 0.00
GCPS disability/interference score	0.86 ± 1.15	0.07 ± 0.26	1.31 ± 1.23	1.50 ± 1.20	0.00 ± 0.00
GCPS disability points	2.03 ± 2.32	1.21 ± 0.56	2.96 ± 2.37	3.33 ± 2.30	0.00 ± 0.00
*GCPS grade*					
I (*n*, %)	38 (52.05)	25 (86.22)	9 (34.62)	4 (22.22)	5 (6.85)
II (*n*, %)	10 (13.70)	2 (6.89)	4 (15.38)	4 (22.22)	0 (0.00)
III (*n*, %)	11 (15.07)	2 (6.89)	5 (19.23)	4 (22.22)	0 (0.00)
IV (*n*, %)	14 (19.18)	0	8 (30.77)	6 (33.34)	0 (0.00)
Current TMD pain VAS	4.62 ± 2.53	3.10 ± 2.04	4.92 ± 2.24	6.61 ± 2.15	0.04 ± 0.26
Worst TMD pain VAS	5.63 ± 3.20	3.48 ± 2.56	6.92 ± 2.68	7.22 ± 2.96	0.14 ± 0.61
Average TMD pain VAS	5.05 ± 2.66	3.14 ± 1.88	6.04 ± 2.27	6.72 ± 2.40	0.08 ± 0.36
Neck pain (*n*, %)	35 (47.95)	5 (17.24)	18 (69.23)	12 (66.67)	6 (8.22)
JFLS‐20 global score	2.80 ± 2.38	1.50 ± 1.59	3.83 ± 2.13	3.42 ± 2.88	0.17 ± 0.35
JFLS‐20 mastication	2.87 ± 2.55	1.72 ± 1.91	3.59 ± 2.28	3.68 ± 3.19	0.27 ± 0.60
JFLS‐20 vertical jaw mobility	3.71 ± 2.99	2.15 ± 2.34	5.04 ± 2.53	4.32 ± 3.49	0.21 ± 0.55
JFLS‐20 verbal and emotional expression	1.83 ± 2.42	0.64 ± 1.08	2.87 ± 2.65	2.26 ± 2.88	0.02 ± 0.08
GAD‐7	7.04 ± 5.41	5.72 ± 5.44	7.77 ± 4.31	8.11 ± 6.56	6.22 ± 4.25
None (*n*, %)	24 (32.88)	14 (48.28)	4 (15.38)	6 (33.34)	32 (43.83)
Mild (*n*, %)	27 (36.98)	8 (27.59)	14 (53.85)	5 (27.77)	22 (30.14)
Moderate (*n*, %)	14 (19.18)	5 (17.24)	6 (23.08)	3 (16.67)	16 (21.92)
Severe (*n*, %)	8 (10.96)	2 (6.89)	2 (7.69)	4 (22.22)	3 (4.11)
PHQ‐15	8.37 ± 5.81	5.76 ± 5.46	11.81 ± 4.93	12.06 ± 7.27	6.00 ± 3.87
None (*n*, %)	20 (27.40)	15 (51.73)	2 (7.69)	3 (16.67)	27 (36.99)
Mild (*n*, %)	20 (27.39)	8 (27.59)	8 (30.77)	4 (22.22)	31 (42.47)
Moderate (*n*, %)	17 (23.29)	3 (10.34)	8 (30.77)	6 (33.34)	13 (17.80)
Severe (*n*, %)	16 (21.92)	3 (10.34)	8 (30.77)	5 (27.77)	2 (2.74)

*Note:* Continuous variables are expressed as means ± standard deviations; categorical variables are expressed as percentages.

Abbreviations: CPI = characteristic pain intensity; GAD‐7 = General Anxiety Disorder‐7; GCPS = Graded Chronic Pain Scale Version 2.0; JFLS‐20 = Jaw Functional Limitation Scale‐20; PHQ‐15 = Patient Health Questionnaire‐15.

TMD patients with local myalgia reported a GCPS CPI of 32.03 ± 18.85, patients with myofascial pain of 59.08 ± 22.00, and patients with myofascial pain with referral of 68.33 ± 24.17.

Interesting, the GCPS pain days were 96.04 ± 65.50 in myofascial pain and 114.39 ± 79.71 in myofascial pain with referral. Moreover, only myofascial pain with referral reported moderate average TMD pain levels (average TMD VAS of 6.04 ± 2.27 and 6.72 ± 2.40, respectively), whereas local myalgia patients reported mild average TMD pain (3.14 ± 1.88). Interesting, more than half of myofascial pain patients (68.18%) reported neck pain.

The GCPS CPI was significantly higher in TMD patients (all subtypes) compared to controls (all *p* < 0.001). Moreover, GCPS CPI was significantly lower in local myalgia compared to either myofascial pain or with referral (all *p* < 0.001). On the other hand, the GCPS CPI was not significantly different in myofascial pain compared to referral (*p* = 0.198). Similar results were obtained when the TMD pain was assessed. See Table 4 for further details.

**TABLE 4:** ANOVA Post Hoc comparisons among temporomandibular disorders groups.

			GCPS.CPI	Average TMD pain VAS	JFLS‐20 global score	GAD‐7	PHQ‐15
			*p*_tukey_	*p*_tukey_	*p*_tukey_	*p*_tukey_	*p*_tukey_
Control	vs	Local myalgia	< 0.001*	< 0.001*	< 0.001*	0.966	0.996
		Myofascial pain with referral	< 0.001*	< 0.001*	< 0.001*	0.449	< 0.001*
		Myofascial pain	< 0.001*	< 0.001*	< 0.001*	0.5	< 0.001*
Local myalgia	vs	Myofascial pain with referral	< 0.001*	< 0.001*	< 0.001*	0.358	< 0.001*
		Myofascial pain	< 0.001*	< 0.001*	< 0.001*	0.402	< 0.001*
Myofascial pain with referral	vs	Myofascial pain	0.198	0.47	0.815	0.996	0.998

*Note:* Significance: *p < 0.001. To identify temporomandibular disorders groups comparison, an ANOVA post hoc test Tukey's HSD was performed, to compare the means of all possible pairs of groups, while adjusting for the multiple comparisons. p‐tukey value adjusted for comparing a family of four estimates.

Results from the present study revealed a mean JFLS‐20 global score of 2.80 ± 2.38 for the total sample. Specifically, it was 1.50 ± 1.59 in local myalgia, 3.83 ± 2.13 in myofascial pain, and 3.42 ± 2.88 in myofascial pain with referral (Table 3). The JFLS‐20 global score was significantly different in TMD patients (all subtypes) compared to controls (all *p* < 0.001). Significant differences were reported in local myalgia compared to either myofascial pain and with referral (all *p* < 0.001), not significant in myofascial pain compared to referral (*p* = 0.815).

Interesting to note, no significant differences were reported in terms of GAD‐7 among groups (all *p* > 0.05).

In terms of PHQ‐15, a score of 8.37 ± 5.81 was reported for the total sample. Specifically, it was 5.76 ± 5.46 in local myalgia, 11.81 ± 4.93 in myofascial pain, and 12.06 ± 7.27 in myofascial pain with referral (Table 3). No significant differences were reported in local myalgia compared to controls (*p* = 0.996) and in myofascial pain compared to referral (*p* = 0.998).

**FIGURE 1:**
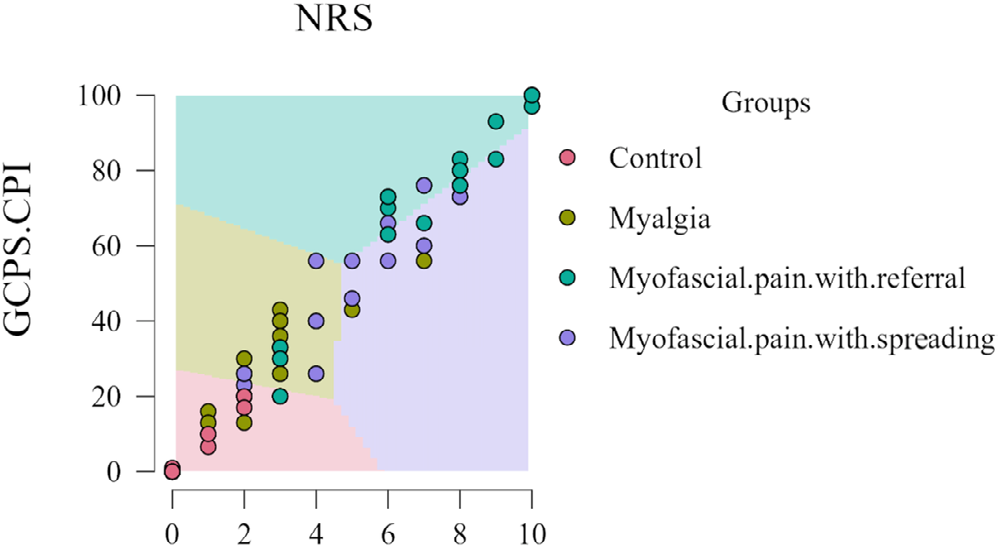
Decision boundary map based on GCPS‐CPI and average TMD pain.

Regarding pain assessment via decision boundary maps, Figure 1 showed that patients with myofascial pain reported the higher levels of both GCPS‐CPI and average TMD pain.

**FIGURE 2:**
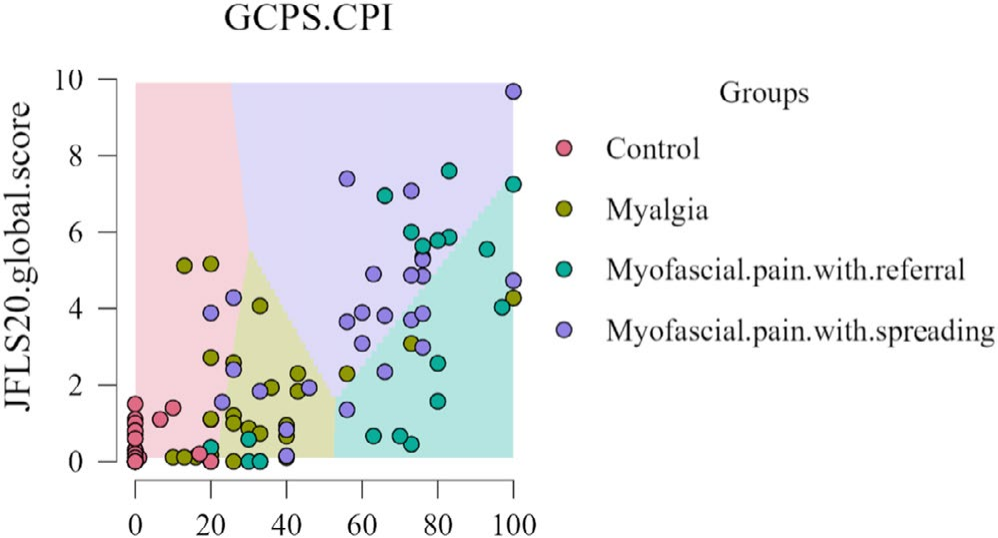
Decision boundary map based on GCPS‐CPI and JFLS‐20 global score.

For moderate pain levels (GCPS‐CPI: 2–6), the JFLS‐20 scale effectively distinguishes between myalgic pain and myofascial pain. Indeed, a JFLS‐20 score around 4 indicates a predictive index for myalgia, whereas a JFLS‐20 score above 5 suggests a myofascial pain pattern (see Figure 2 for further details).

**FIGURE 3:**
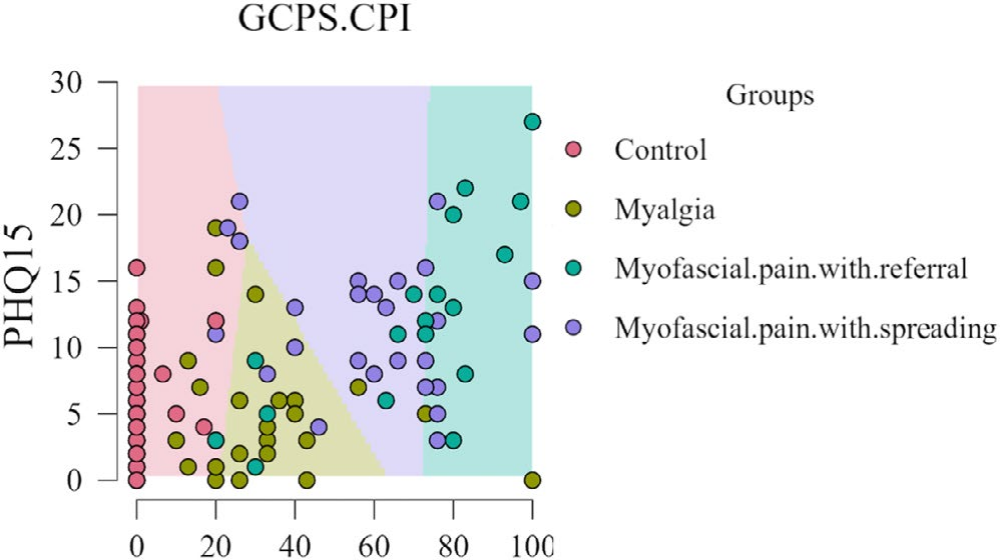
Decision boundary map based on GCPS‐CPI and PHQ‐15.

Lastly, the PHQ‐15 showed similar expected results and decision boundary maps as the JFLS‐20 in relation to the GCPS‐CPI (Figure 3).

## 4 Discussion

During the last decades, scientific literature has shown that TMD etiology may involve both pathophysiological and psychosocial factors. However, very few studies have investigated the prevalence of general physical functioning and pain intensity, as well as limitations, depression and anxiety, and physical symptoms in patients with different myalgia diagnoses.

The present study on TMD data from Southern Italy aimed to assess a potential association between DC/TMD Axis I myalgia patients and Axis II assessment and to evaluate the psychosocial predictors of high pain‐related disability, whose assessment should be beneficial throughout clinical decision‐making progressions.

As reported by Michelotti et al. (Michelotti et al. 2016) optimal diagnostic criteria should comprise efficient tools that could identify at an early stage the patients at risk of developing chronic pain conditions, thus enabling an early intervention. The most efficient clinical diagnosis process should integrate the data collected by the Axis I clinical examination with the psychosocial evaluation available through the DC/TMD questionnaires to achieve a true multidimensional assessment according to the biopsychosocial model. However, it is important to administer the least number of the most efficient questionnaires, avoiding the subject burden and fatigue (Michelotti et al. 2016).

In the present study, the DC/TMD Axis II instruments were used to assess general physical functioning (GCPS) and pain intensity (CPI), limitations (JFLS‐20), depression and anxiety (GAD‐7), and physical symptoms (PHQ‐15) to evaluate the TMD‐related biopsychosocial model.2.

The final cohort involved 73 people with myogenous pain TMD‐related with a median age of 39 years and a male to female ratio of 1.7, which was slightly lower than those reported by previous research conducted in other populations (Wieckiewicz et al. 2014; Yildiz et al. 2023). The disproportion of female (63%) compared to male (37%) suggests that women in the Southern Italy population are more susceptible to developing TMD than men. Moreover, these findings corroborated previous studies, demonstrating the contributory roles of sex in the pathophysiology of TMD. In particular, the etiopathology of TMD is considered multifactorial, with some predisposing or perpetuating factors (Shaefer et al. 2013); among them, gender plays a role in the pathophysiology of various chronic pain conditions and is considered the biggest risk factor in the development of TMD and orofacial pain (Shaefer et al. 2013). Furthermore, females tend to show more symptoms and seek treatment more often than males; this is confirmed by the scientific literature, reporting a gender difference in pain thresholds, temporal summation, pain expectations, and somatic awareness (Shaefer et al. 2013; Osborne and Davis 2022).

The included masticatory myalgia patients reported moderate pain levels: current pain VAS of 4.62, worst pain VAS of 5.63, average pain VAS of 5.05. Specifically, patients with local myalgia reported a GCPS CPI of 32.03 ± 18.85, patients with myofascial pain of 59.08 ± 22.00, and patients with myofascial pain with referral of 68.33 ± 24.17. Manfredini et al. (Manfredini et al. 2009) supported the existence of a close association between pain and psychosocial disorders in TMD patients, and our findings mirror previous studies that showed how myofascial pain patients scored higher on depression and somatization tests (Yap et al. 2002; Canales et al. 2019; Manfredini, Borella, et al. 2010).

The somatization can be considered as the tendency to experience non‐specific physical symptoms and somatic distress in response to psychosocial stress and the subsequent request for medical help (Lipowski 1988).

A systematic review by Zijlema et al. (Zijlema et al. 2013) identified and described 40 self‐report questionnaires for somatic symptoms and recommended the PHQ‐15 and the somatization subscale of the Symptom Checklist 90‐item version (SCL‐90 SOM) as the best options for evaluating pain of unknown origin, nausea and vomiting, palpitations, tendency to faint, shortness of breath, tremor of the limbs, abdominal pain, and several other ailments (Von Korff et al. 1992).

In the present study, the PHQ‐15 was used for evaluating the non‐specific physical symptoms and for assessing comorbidities and overall symptom reporting. Our study showed a high level of somatization in patients affected by temporomandibular myofascial pain. Specifically, 45.20% of the included patients reported a moderate or severe PHQ‐15 score, and a mean score of 8.37 was reported for the total sample. The higher mean scores of PHQ‐15 were reported for myofascial pain with referral (12.06 points) and for myofascial pain (11.81 points), and significant differences between myofascial pain and local myalgia patients (*p* < 0.001) were reported.

An interesting study by Hietaharju et al. (Hietaharju et al. 2021) analyzed 197 TMD pain patients who were categorized into TMD subtypes 1, 2, and 3 (GCPS I/II‐low; II‐high; III/IV, respectively) based on their biopsychosocial profiles according to the GCPS questionnaire. The authors showed that subtype 3 patients reported the higher score of physical symptoms (PHQ‐15) and anxiety (GAD‐7).

This was in line with our results that revealed a high score of PHQ‐15 for myofascial pain patients, who reported the higher number of subjects with GCPS grade III/IV (52.27%).

Moreover, results from the present study revealed that patients affected by myofascial pain with referral reported the higher score of: GCPS CPI (68.33 points), current pain VAS (6.61 points), worst pain VAS (7.22 points), average pain VAS (6.72 points), JFLS‐20 mastication (3.68 points), GAD‐7 (8.11 points), and PHQ‐15 (12.06).

The somatic symptoms are strictly associated with comorbid psychiatric disorders such as depression and anxiety (Von Korff et al. 1992; Spitzer et al. 2006), which were reported to be the most pronounced symptoms in patients with chronic TMD (Reiter et al. 2015).

A strict relationship between PHQ‐15 and GAD‐7 was shown by Kuć et al.34, who reported anxiety disorders of various intensity in 44% of the analyzed subjects with myofascial pain with referral.

Results from the present study revealed that 67.12% of included patients reported anxiety or depression symptoms, with a mean GAD‐7 score of 7.04 for the total sample. However, 56.16% of controls reported mild to severe GAD‐7 scores, and no significant differences were reported either between TMD patients and controls or among TMD subtypes.

Our results were not in line with Simoen et al. (Simoen et al. 2020) who compared the levels of depression and anxiety of a group of TMD patients with the general population and showed statistically significant higher scores for GAD‐7 in the study group, suggesting that screening for depression and anxiety should be considered during the clinical assessment in TMD patients.

The JFLS‐20 provides a global measure of functional limitation of the masticatory system in terms of mastication, vertical jaw mobility, and emotional and verbal expression. A significant relationship between oral and general health status and jaw functional limitation scores was reported, and authors found that the health status worsened when JFLS scores increased (Oghli et al. 2019).

Results from the present study revealed a mean JFLS‐20 global score of 2.80 for the total sample. Interestingly, patients with myofascial pain reported the higher mean score of JFLS‐20 mastication, JFLS‐20 vertical jaw mobility, and JFLS‐20 verbal and emotional expression. Moreover, the JFLS‐20 global score was significantly different in TMD patients (all subtypes) compared to controls (all *p* < 0.001).

A study by Kuć et al. (Kuć et al. 2021) found a JFLS‐20 global score of 1.61 points and a mean value of JFLS‐20 mastication of 1.93 points, and concluded that the limitations in mastication, vertical jaw mobility, and verbal and emotional communication were significant predictors of craniomandibular dysfunction. It is noteworthy that our results were slightly worse, as we found a JFLS‐20 global score of 2.80 and a mean value of JFLS‐20 mastication of 2.87.

A recent study by Micarelli et al. (Micarelli et al. 2022) showed a significant inversely proportional relationship between JFLS‐20 and cervical range of motion in patients with TMD, and the authors suggested the existence of a direct link between TMD and cervical spine impairment. Our findings showed that 47.95% of myogenous TMD patients reported neck pain and 34.25% reported headache attributed to TMD.

In the scientific literature, authors reported that subjects with masticatory myofascial pain were shown to suffer from greater neck disability than asymptomatic controls. Moreover, a higher neck disability was shown to be correlated with greater sternocleidomastoid, anterior temporalis, and trapezius muscle sensitivity (Liang et al. 2019) and a strict relationship among neck pain, TMD, and headache was reported (Ferrillo et al. 2023).

Interestingly, a recent study by Tchivileva et al. (Tchivileva et al. 2021) evaluated subjects suffering from TMD and headache and distinguished the presence of headache attributed to TMD in people with myogenous TMD. The authors found that 61.6% had headache attributed to TMD, thus showing a higher prevalence than those reported in the present study (34.25%). However, the authors pointed out that the distinction among muscular TMD, primary headache, and headache attributed to TMD remains elusive, as an extensive overlap of symptoms has been reported in many cross‐sectional studies (Svensson 2007).

The above‐mentioned data revealed that myofascial TMD patients reported high levels of pain intensity, functional limitations, and physical symptoms, and that DC/TMD Axis II should be applied to identify unexplained somatic symptoms and to monitor changes in their severity to provide a comprehensive and interdisciplinary treatment plan (Dworkin et al. 2002).

Despite employing the evidenced‐based DC/TMD methodology, this study is not free from limitations. First, the relatively small number of subjects in each subgroup might have affected the power of the analysis, which should be interpreted with caution. Second, the research focused only on Southern Italy TMD patients; thus, the findings should be validated among different racial groups before being generalized. Third, more than half of the patients (64.38%) reported arthralgia. Thus, an overlapping between arthrogenous and myogenous symptoms could have affected the study results.

## 5 Conclusions

Taken together, findings of this study showed that myofascial pain patients reported the significantly higher levels of pain intensity, functional limitations, and physical symptoms, compared to both local myalgia and control patients.

The above‐mentioned data suggest that DC/TMD Axis II should be applied to evaluate emotional status and to identify unexplained somatic symptoms and to monitor changes in their severity, to provide a comprehensive and interdisciplinary treatment plan. Future research is needed to confirm these data and to provide more information on the temporal/causal relationship between myofascial pain TMD and biopsychosocial factors.

## Author Contributions

**Martina Ferrillo:** conceptualization, investigation, methodology, writing – original draft, data curation. **Nicola Marotta:** data curation, formal analysis, writing – original draft. **Andrea Demeco:** visualization, formal analysis. **Laura Gallelli:** investigation, data curation, visualization. **Maria Teresa Inzitari:** visualization. **Umile Giuseppe Longo:** investigation, visualization. **Francesco Riccitiello:** visualization. **Antonio Ammendolia:** writing – review and editing. **Amerigo Giudice:** writing – review and editing. **Alessandro de Sire:** writing – review and editing.

## Ethics Statement

The study was approved by the Ethical Committee of Calabria Region (protocol number: 61/2025).

## Consent

All participants were asked to read and sign an informed consent form. The study was undertaken in accordance with the Declaration of Helsinki of 1975.

## Conflicts of Interest

The authors declare no conflicts of interest.

## Data Availability

The data that support the findings of this study are available from the corresponding author upon reasonable request.
